# Increase in stress contributes to impaired jaw function in juvenile idiopathic arthritis: a two-year prospective study

**DOI:** 10.1186/s12969-024-00966-4

**Published:** 2024-02-26

**Authors:** Alexandra Dimitrijevic Carlsson, Kerstin Wahlund, Erik Kindgren, Martina Frodlund, Per Alstergren

**Affiliations:** 1 Orofacial Pain and Jaw Function, Malmö University, Malmö, Sweden; 2 Centre for Oral Rehabilitation, Linköping, and Department of Biomedical and Clinical Sciences, Linköping University, Linköping, Sweden; 3 Scandinavian Center for Orofacial Neurosciences, Malmö University, Malmö, Sweden; 4 Department of Orofacial Pain and Jaw Function, Kalmar County Hospital, Kalmar, Sweden; 5 Department of Pediatrics, Västervik Hospital, Västervik, Sweden; 6 Division of Pediatrics, Department of Clinical and Experimental Medicine, Linköping University, Linköping, Sweden; 7 Department of Pediatrics, Skaraborg Hospital, Skövde, Sweden; 8 Rheumatology/Division of Inflammation and Infection, Department of Biomedical and Clinical Sciences, Linköping University, Linköping, Sweden; 9 Skåne University Hospital, Specialized Pain Rehabilitation, Lund, Sweden; 10 Orofacial Pain Unit, Malmö University, Malmö, Sweden

**Keywords:** Juvenile Idiopathic Arthritis, Orofacial pain, Psychological distress, Psychosocial, Stress, Temporomandibular joint disorders

## Abstract

**Background:**

Stress in patients with Juvenile Idiopathic Arthritis (JIA) has been found to be associated with orofacial pain, psychological distress, jaw dysfunction and loss of daily activities in a cross-sectional study. The aim of this study was to investigate the relations between stress and change of stress over time versus changes in orofacial pain, psychosocial factors and jaw function over a two-year period in patients with JIA.

**Methods:**

This is a two-year prospective follow-up study involving 40 JIA patients. At baseline (2015) the median age was 12 years and at two-year follow up (2018) 14 years. The JIA patients were examined clinically and with questionnaires at baseline and follow-up with the diagnostic criteria for temporomandibular disorders (DC/TMD) and completed the same set of DC/TMD questionnaires regarding orofacial pain symptoms and psychosocial factors.

**Results:**

Change in stress was associated with change in catastrophizing, psychological distress as well as limitation in general function and jaw function.

**Conclusions:**

This study emphasizes the importance of maintaining a low stress level in patients with JIA since an increase in stress level over a two-year period seems to impair jaw function as well as psychological distress and catastrophizing.

## Background

Juvenile idiopathic arthritis (JIA) is the most frequently occurring rheumatic disease in children of unknown etiology, that presents in children by the age of 16 years [1]. JIA comprise seven subtypes as defined by the international League of Rheumatology Associations (ILAR). These subtypes are categorized based on number of affected joints and the presence of extra-articular, systemic, or serum symptoms. The subtypes are systemic, psoriatic, oligoarticular (persistent and extensive) polyarticular (RF+ and RF-) and undifferentiated arthritis. Serological biomarkers associated with JIA include anti-nuclear antibodies (ANA), rheumatoid factor(RF) and anti-citrullinated protein antibodies (ACPA) [2]. The etiology and pathogenesis are not fully understood, however both genetic factor and environmental factors contribute the development of JIA [3]. Symptoms include unpredictable patterns of joint inflammation, stiffness, pain and fatigue that can persist into adulthood [4–7]. The disease is also associated to a variety of negative outcomes (pain interference with physical, educational, emotional and social activities) [8–10]. JIA pain also affects patients psychosocially (sense of being misunderstood and stigmatize, overwhelming pain and despair, quality of life and mental health challenges) [11–13].

Structural damage of the temporomandibular joint (TMJ) is a common consequence of TMJ arthritis in JIA [14, 15]. Initially, TMJ arthritis might not show any symptoms, but as the disease advances, patients frequently experience orofacial symptoms including pain, impaired jaw function and a reduced quality of life [16–24]. Diagnosis of TMJ involvement comprise clinical examination of the TMJ, e.g. mouth opening capacity and TMJ pain, as well as instrumental examinations like magnetic resonance imaging (MRI). Early detection of TMJ involvement can increase the possibilities to reduce facial growth anomalies and skeletal malocclusions [25, 26].

Peers of the JIA population may also experience orofacial pain and TMD. TMD is associated with various clinical signs and symptoms involving the masticatory muscles, TMJ and/or their supportive tissues. The prevalence of painful TMD among the adolescent population was reported to be around 4,2 %, based on self-reported pain screening questionnaires. Stress has been identified as a trigger for TMD pain in the adolescent population [27, 28].

In our baseline case-control study, stress was associated with orofacial pain in JIA, which in turn was linked to psychological distress, jaw dysfunction and disruptions in daily activities. The overall inflammatory activity also plays an important role in contributing to orofacial pain in JIA [22]. Previous cross-sectional studies have found an association between arthritis-induced orofacial symptoms and general health-related quality of life using the Childhood Health Assessment Questionnaire (CHAQ) and the Child Health Questionnaires (CHQ) [23, 29]. There is, unfortunately, an obvious lack of knowledge, regarding the development or change of orofacial symptoms over time in JIA. However, persistent long-term consequences of TMJ involvement (symptoms, dysfunctions, and damage of the TMJ) into adulthood has been shown in a 17-year follow-up study [19]. In another study with a two-year follow-up, orofacial pain and functional disability were frequently reported by a group of JIA patients and appeared to persist over time in the majority of patients, significantly impairing their oral health-related quality-of-life [21].

The aim of this study was to investigate the relations between stress and change of stress over time versus changes in orofacial pain, psychosocial factors and jaw function over a two-year period in patients with JIA.

## Methods

### Study design and subjects

This longitudinal prospective study with a two-year follow-up of the JIA patients was conducted at the Centre of Oral Rehabilitation (COR) in Linköping, Sweden. At baseline, patients with JIA were referred from four pediatric departments in South-east Sweden (Linköping university hospital, Vrinnevi Hospital Norrköping, Motala Hospital and Västervik Hospital).

The baseline examinations were carried out 2015 to 2018 and follow-up examinations were performed after two years (2018 – 2021) [22]. The median (25/75^th^ percentiles) interval between the baseline and follow-up examinations was 31 (26/35) months. All patients from the baseline study were invited to participate in the two-year follow-up study. Forty JIA patients (30 girls and 10 boys) aged 6 to 16 years were included, at baseline the median age was 12 (9/14) years and at follow-up 14 (12/16) years. Inclusion and exclusion criteria at baseline are shown in Table 1.

**Table 1:** Inclusion and exclusion criteria at baseline

**INCLUSION CRITERIA**	**EXCLUSION CRITERIA**
• Age 6-16 years	• Diabetes
• JIA-diagnosis according	• Inflammatory bowel disease
to ILAR criteria	• Other chronic pain condition than JIA
	• Psychiatric disease (depression and anxiety were allowed due to the frequent occurrence and important role in chronic pain)

*ILAR* International League of Associations for Rheumatology

Demographic data as well as disease activity, medication and DC/TMD diagnoses are reported in Table 2. JIA subdiagnoses, according to International League of Association for Rheumatology (ILAR) criteria [2] in our patient sample were 18 (45%) with oligoarthritis, 13 (33%) with polyarthritis and 9 (22%) with other subtypes of JIA (systemic arthritis, psoriatic arthritis, enthesitis-related arthritis or undifferentiated arthritis). The patient sample accurately mirrored the JIA patient population in Sweden [30].

**Table 2:** Demographic data, disease activity and temporomandibular disorder diagnoses for 40 patients with juvenile idiopathic arthritis at baseline and 40 patients at two year follow- up

			BASELINE					TWO-YEAR FOLLOW UP			
			**Percentiles**					**Percentiles**			
		**Median**	25th	75th	% pos	n	**Median**	25th	75th	% pos	n
**Individuals**											
Age	years	12	9	14		40	14	12	16		40
Sex	boys/girls					10/30					10/30
Age at diagnosis	years	9	4	11		40	9	4	11		40
Disease duration	years	4	2	7		40	6	3	8		40
**Disease activity**											
JADAS71	0-101	6.2	3.1	9.9		40	3.0	0.5	8.5		40
Erythrocyte sedimentation rate^a^	mm	7	4	10	8	39	0	0	0	0	39
C-reactive protein^a^	mg/L	0	0	0	5	39	0	0	0	3	39
Rheumatoid factor	IU/mL	0	0	0	7	39	0	0	0	8	39
Anti-citrullinated antibodies	U/mL	0	0	0	10	40	0	0	0	10	40
Antinuclear antibodies					35	40				35	40
HLA-B27 pos					12	40				12	40
**Medication**											
NSAID					60%	24				43%	17
DMARD					63%	25				38%	15
Glucocorticoids					15%	6				7%	3
Biologics					27%	11				35%	14
Biologics and DMARD					13%	5				15%	6
No medication					10%	4				17%	8
**DC/TMD diagnoses**											
Myalgia	n					8					10
Myofascial pain with referral	n					2					1
Arthralgia	n (joints)					7					11
Headache attributed to TMD	n					3					1
Combinations from above											
Myalgia and arthralgia	n					2					6
Myalgia, arthralgia and headache	n					2					1

*n* number of observations, *JADAS71* 71-joint Juvenile Arthritis Disease Activity Score, *DC/TMD* Diagnostic criteria for temporomandibular disorders. *NSAID* Non-steroidal anti-inflammatory drug, *DMARD* Disease-modifying anti-rheumatic drugs (Methotrexate, Plaquenil, Orencia, Salazopyrin), Glucocorticoids (Prednisolone) Biologics at baseline (Adalimumab (4), Etanercept (5), Abatacept (1) and Golimumab (1)) and for 2-year follow up (Adalimumab (7), Etanercept (2), Tocilizumab (3) and Infliximab (1))

^a^Normal values for ESR (<30 mm/h), CCP (<7 U/mL) and CRP (<5 mg/L) were counted as 0. Other subtypes of JIA (systemic arthritis, psoriatic arthritis, enthesitis-related or undifferentiated arthritis)

JIA patients with newly diagnosed JIA are regularly referred from the pediatric departments in Östergötland County, to the COR, for yearly screening of their TMJs regardless of inflammatory activity. The JIA patient in this study at baseline and 2-year follow- up that was diagnosed with a TMD diagnosis and received treatment at the COR.

### Clinical examination

The study participants were examined and diagnosed according to the diagnostic criteria for temporomandibular disorders (DC/TMD) at baseline and at two-year follow-up [31]. The DC/TMD is structured into two domains: Axis I, which focuses on clinical conditions related to physical health, and Axis II, which assesses factors related to psychosocial distress. The DC/TMD has previously been applied in studies involving JIA children aged 4-16 years and the findings have been published and presented [24, 32]. Additionally, the DC/TMD clinical examination has been utilized in studies involving healthy children experiencing TMD pain, ranging from ages 7 to 19 years [33–36].

The DC/TMD Axis I diagnoses are derived from a combination of a pain history, assessed by a questionnaire, and a well-defined and structured clinical examination. The clinical examination assesses familiar pain localizations, jaw movement capacity (lateral, protruding, and mouth opening), familiar jaw movement pain, TMJ noises and familiar pain upon palpation of the masticatory muscles and TMJ. The criteria for DC/TMD Axis I diagnoses are validated from 18 years of age and comprise TMJ arthralgia, masticatory muscle myalgia, headache attributed to TMD, degenerative joint disease and TMJ disc displacements. Multiple diagnoses are allowed in DC/TMD [31]. DC/TMD Axis II evaluates the patient's psychosocial function, distress, and pain-related disability by validated instruments (questionnaires) and interpretation guidelines. These instruments assess pain intensity, pain-related disability, psychological distress and jaw function. One orofacial pain specialist (ADC) conducted all examinations and was pre-trained and calibrated in the clinical and research application of DC/TMD by the DC/TMD Training and Calibration Center at the Department of Orofacial Pain and Jaw Function, Faculty of Odontology, Malmö University, Sweden. All JIA patients at the baseline and at the two-year follow-up examination completed the questionnaires before clinical examination. Twenty-three study individuals, 12 – 16 years of age answered all questions. The questions regarding stress and catastrophizing were not answered by children younger than 12 years of age due to the unknown validity of these questionnaires for that age group.

### Psychosocial status

The following psychosocial aspect were assessed: pain intensity, pain-related disability, pain location(s), jaw functional limitations, anxiety, depression, catastrophizing functional ability and disease activity were evaluated. These questionnaires were used to assess these aspects: Graded Chronic Pain Scale (GCPS) [37], Jaw Function Scale (JFLS -8) [38], Patient Health Questionnaire (PHQ–4) [39], Pain Catastrophizing Scale (PCS) [40], Perceived Stress Scale (PSS-10) [41], Body Pain Drawings Locations [42], Childhood Health Assessment Questionnaire (CHAQ) [43] and Juvenile Arthritis Disease Activity Score-71 (JADAS-71) [44].

The questions in the PHQ-4 (14-16 years), PCS (8–17 years), JADAS and CHAQ has been validated in children and adolescents [43–46]. The questions in GCPS (12-19 years), JFLS-8 (6-16 years) and PSS (12-18 years) has been used in previous studies in children and adolescents [22, 47, 48]. For a full description of the assessment of these psychosocial factors, see Dimitrijevic Carlsson et al. 2019 [22].

### Statistical analyses

Non-parametric statistics were used. For descriptive statistics median and 25^th^/75^th^ percentile were reported. The Wilcoxon’s ranked test was used to calculate the significance of individual change over time. For analytical statistics the Spearman rank correlation coefficient was used to calculate the significance of correlations between variables: change over time versus change over time as well as baseline versus change over time. A probability level of *p* < 0.05 was considered significant. All statistical analysis were performed using Stata 15.1 Special Edition software (StataCorp, College Station, TX, USA).

## Results

The baseline clinical and psychosocial variables are reported in Tables 3 and 4, as are the changes in variables over the two-year interval.

**Table 3:** Clinical variables at baseline and change in these variables over a two-year period in 40 patients with juvenile idiopathic arthritis

		**Baseline**				**Changes over two years**			
			**Percentile**				**Percentile**		
		**Median**	25th	75th	n	**Median**	25th	75th	n
Maximum mouth opening without pain	*mm*	38	30	43	40	3	-3	8	40
Maximum voluntary mouth opening	*mm*	47	44	52	40	4	1	6	40
Pain in TMJs on maximum mouth opening	*0-1*	0	0	0	40	0	0	0	40
Pain in muscles on maximum mouth opening	*0-1*	0	0	0	40	0	0	0	40
Number of painful jaw movements	*0-4*	0	0	0	40	0	0	0	40
Palpation pain, TMJ	*0-1*	0	0	0	40	0	0	0	40
Palpation pain, masticatory muscles	*0-1*	0	-1	0	40	0	-1	0	40

*n* Number of observations, *TMJ* Temporomandibular joint

**Table 4:** Psychosocial variables at baseline and change in these variables over a two-year period in 40 patients with juvenile idiopathic arthritis. Solely 23 patients (12-16 years of age) answered the questionnaire regarding depression, catastrophizing and stress

	**Baseline**				**Changes over two years**			
		**Percentile**				**Percentile**		
	**Median**	25:e	75:e	n	**Median**	25:e	75:e	n
CHAQ	0.25	0.00	0.50	40	0.00	-0.25	0.00	40
JADAS71	6.2	2.9	10.1	40	-3.1	-6.8	-0.9	40
Characteristic pain intensity	0	0	3	40	0	0	1.2	40
Pain-related disability	0	0	0	40	0	0	0	40
Depression	0	0	3	23	0	-1	0	23
Stress	10	2	20	23	-0.5	-11	0	23
Catastrophizing	9	3	16	23	-1	-10	0	23
Jaw function limitation	0	0	3	40	0	-3	0	40

*n* Number of observations, *CHAQ* Cchildhood Health Assessment Questionnaire, *JADAS71* Juvenile Arthritis Disease Activity Score based on 71-joint count, *Characteristic pain intensity* Subscale of the Graded Chronic Pain Scale, *Pain-related disability* Subscale of the Graded Chronic Pain Scale, *Depression* Assessed with Patient Health Questionnaire, *Stress* Asses with Perceived Stress Scale, *Catastrophizing* Assessed by Pain Catastrophizing Scale and jaw function limitation: was assessed by Jaw Function Limitation Scale

The significant relations between changes over time in clinical and psychosocial variables are shown in Table 5 and the significant relations between baseline stress levels versus changes in these variables are shown in Table 6.

**Table 5:** Significant correlations between changes over time in stress versus change over time in clinical and psychosocial variables over two years in 23 patients with juvenile idiopathic arthritis (12-16 years of age)

		**Correlation**		
**Change in**	**Change in**	rs	n	*P*
Stress	Catastrophizing	0.66	23	0.001
Stress	Psychological distress	0.76	23	< 0.001
Stress	Jaw Function Limitation	0.69	23	< 0.001
Stress	CHAQ	0.42	23	0.045

*rs* Rank-sum, *n* Number of observations, *P* Probability

**Table 6:** Significant correlations between stress at baseline versus change over time in clinical and psychosocial variables over two years in 23 patients with juvenile idiopathic arthritis (12-16 years of age)

		**Correlation**		
**Baseline**	**Change in**	rs	n	*P*
Stress	Number of painful movements	-0.55	23	0.006
Stress	CHAQ	-0.42	23	0.044
Stress	Psychological distress	-0.77	23	<0.001
Stress	Catastrophizing	-0.66	23	<0.001
Stress	Jaw Function Limitation	-0.69	23	<0.001
JADAS	Stress	-0.35	23	0.025
Orofacial pain intensity	Stress	-0.50	23	0.001
Pain-related disability	Stress	-0.55	22	0.008
Psychological distress	Stress	-0.76	23	< 0.001
Catastrophizing	Stress	-0.66	23	0.006

*rs* Rank-sum, *n* Number of observations,* P* Probability

**Fig. 1:**
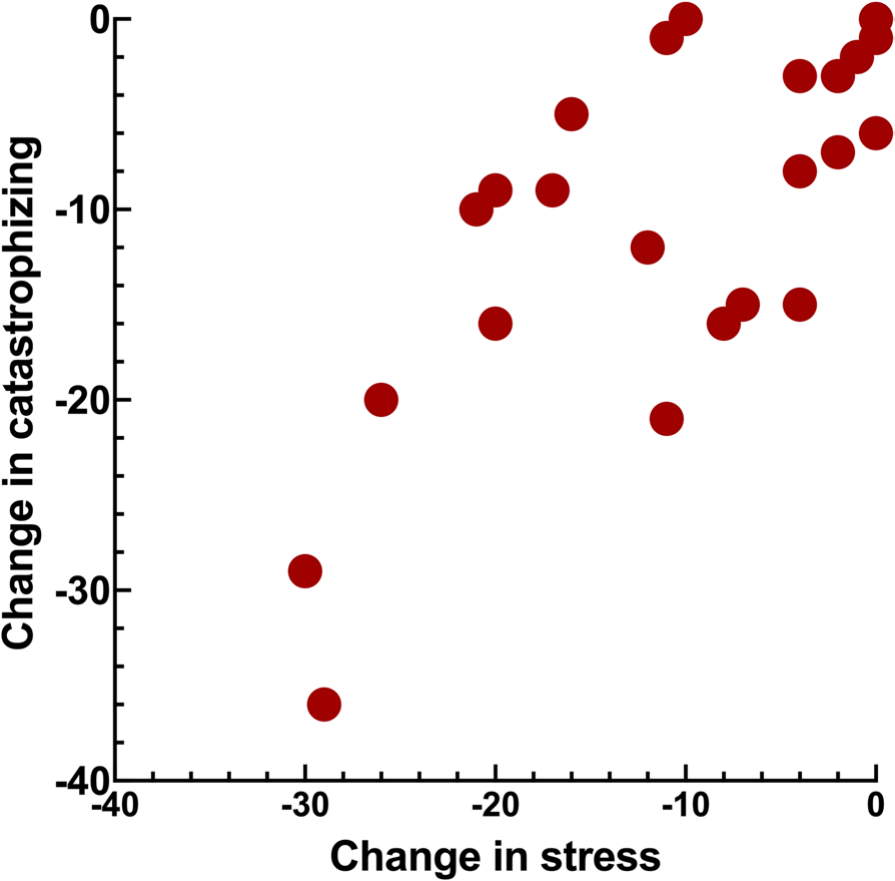
Scatter-plot showing the association between change in stress and change in catastrophizing (*r*_s_ = 0.66, *n* = 23, *p* = 0.001) in 23 JIA patients after a two-year follow-up interval

**Fig. 2:**
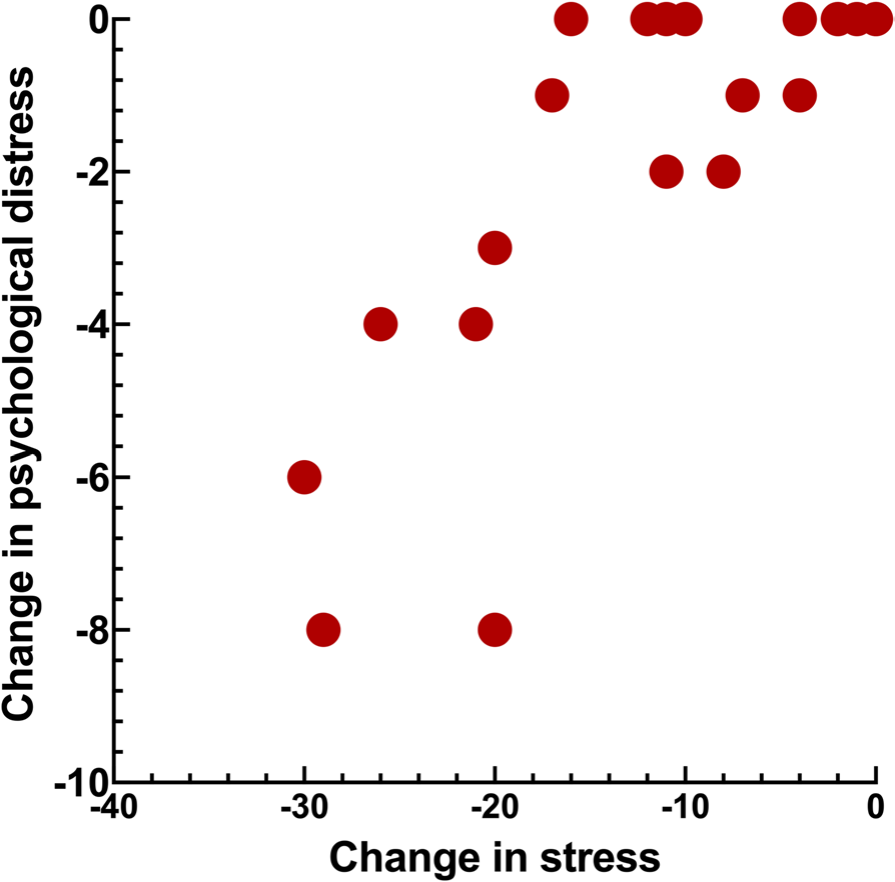
Scatter-plot showing the association between change in stress and change in psychological distress (*r*_s_ = 0.76, *n* = 23, *p* < 0.001) in 23 JIA patients after a two-year follow-up interval

**Fig. 3:**
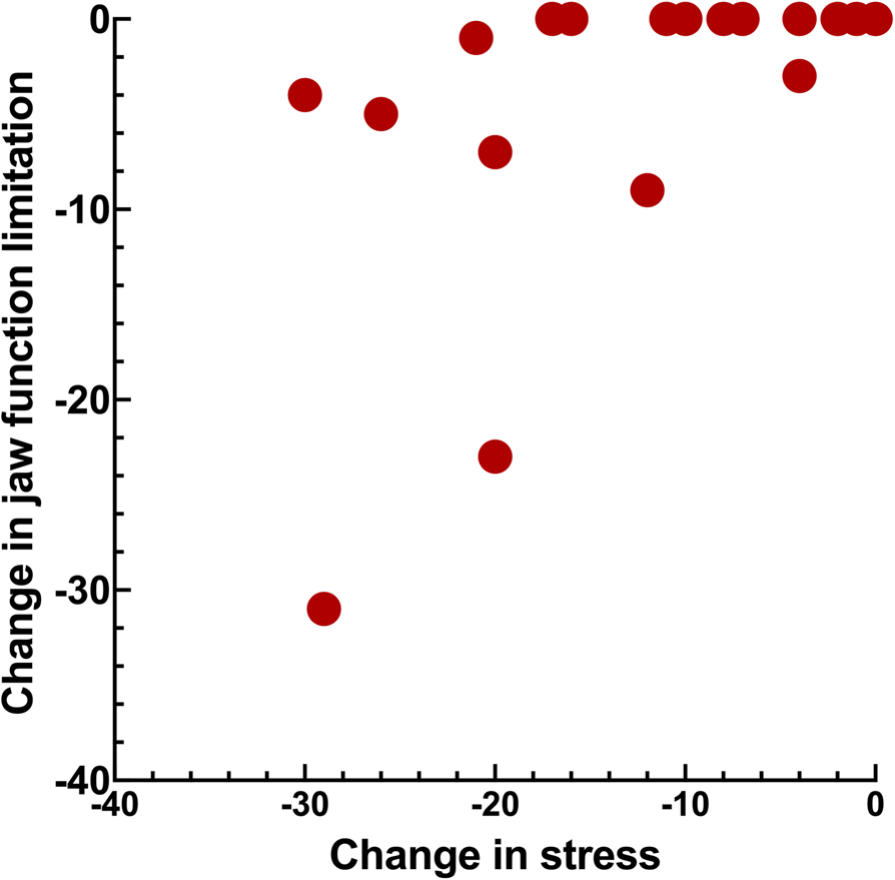
Scatter-plot showing the association between change in stress and change in jaw function limitation (*r*_s_ = 0.69, *n* = 23, *p* < 0.001) in 23 JIA patients after a two-year follow-up interval

**Fig. 4:**
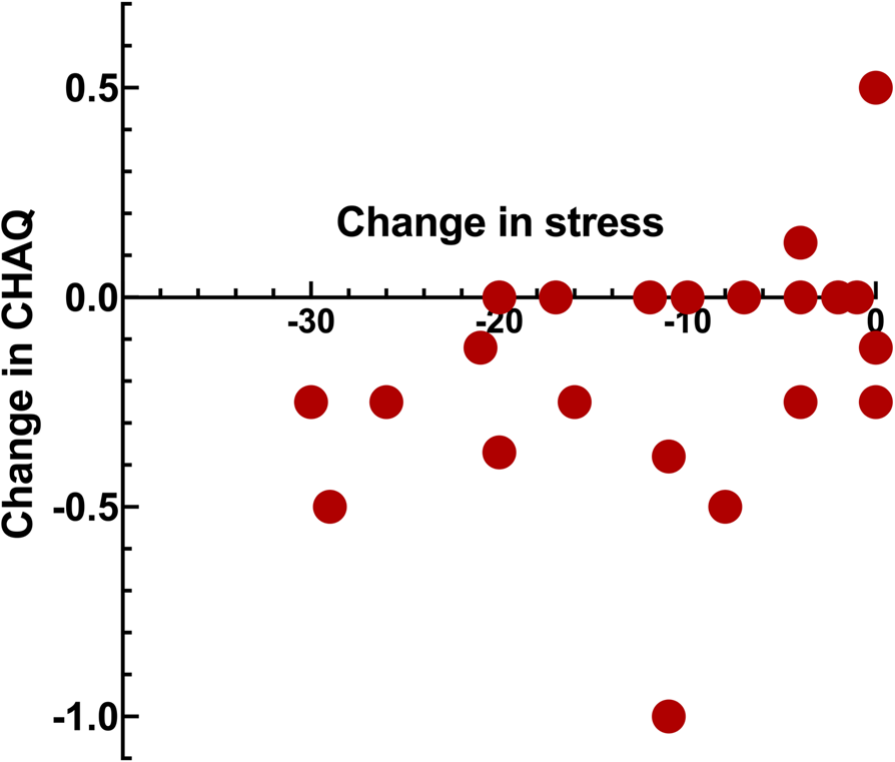
Scatter-plot showing the association between change in stress and change in general functional ability, as catastrophizing (*r*_s_ = 0.66, *n* = 23, *p* = 0.001) in 23 JIA patients after a two-year follow-up interval

Figures 1, 2, 3 and 4 shows the significant relations between change in stress versus psychosocial and functional variables. Change in stress was positively correlated to change in catastrophizing, psychological distress, jaw function limitation and CHAQ (Tables 4 and 5, Figs. 1, 2, 3 and 4).

## Discussion

This study emphasizes the importance of maintaining a low stress level in patients with JIA. In our study, increase over time in stress was associated with impaired jaw function and an increase in catastrophizing. Self-reported orofacial pain and functional disability were common findings in our cohort of JIA patients followed over two years. Symptoms seem to persist over time in most patients and have a significant negative impact on oral health-related quality-of-life [21].

In a longitudinal study with a follow-up interval of one year in JIA patients, TMJ symptoms were mild and fluctuated during the study period and the most common symptom was pain on jaw function [49]. JIA patients have been shown to have a decreased jaw function and a lower maximal bite force, compared to healthy children [50]. Also, JIA children have a significantly lower physical activity level and lower muscle strength compared to their healthy peers [51]. Psychological stress, which is not uncommon among JIA patients and related to the disease [52], in the form of anxiety and depression can increase oral parafunctions including bruxism and limit jaw function. On the other hand, bruxism and other oral parafunctions may also be a sign of psychological stress [53]. Although the mechanisms by which stress may affect jaw function are unknown, it may be mediated via the relation found between changes in stress over time versus change in catastrophizing over time and/or change in psychological distress over time. In a study by Fair and colleges, 23% of the JIA children reported moderate to severe symptoms of anxiety and depression and these symptoms were associated with pain and stress [54]. Other studies have reported similar results regarding the relation between pain, stress and mental health symptoms [12, 55]. Catastrophizing may in turn negatively affect kinesiophobia, which hinder physical performance and functional quality-of-life in JIA [56]. In a recent two-year follow-up JIA study assessing the impact of psychosocial stress factors on physical activity observed that decreased physical activity was associated with higher disease activity and higher disease-specific psychosocial stress [57]. Catastrophizing has been shown to be independently associated with pain severity, disability and somatic complaints in school children and children with chronic pain [58]. Higher stress may thereby increase catastrophizing and psychological distress which, in turn, may influence kinesiophobia [59].

In the present study we also found that increased stress during the two-year follow-up interval was associated with a reduced general functional ability in daily activities, as assessed with CHAQ. These limitations may have severe impact on school attendance and participation in sports activities [60].

Taken together, our findings support a relation between stress, orofacial pain, mood and orofacial function and emphasizes the importance of early assessment and intervention of higher stress levels when examining and treating JIA patients from an orofacial aspect.

Baseline stress level was negatively associated with number of painful movements, CHAQ, psychological distress, catastrophizing and jaw function limitation. In turn, baseline levels of JADAS, orofacial pain intensity, pain-related disability, psychological distress and catastrophizing were negatively related to change in stress over time. We consider these relations to be a regression-towards-the-mean effect since there is no plausible or relevant biological explanations.

### Methodological considerations

This study is the first longitudinal study of JIA patients based on the results from a cross-sectional study where stress was shown to be associated with orofacial pain, mood and jaw function [22, 60]. The current study retained 89% of the patients from the cross-sectional study and followed these for two years. To our knowledge, this is the first two-year prospective study to incorporate the use of DC/TMD including the clinical condition and psychosocial factors in children with JIA. All clinical examinations were performed by the same operator, an operator trained and calibrated in the clinical and research use of DC/TMD. Likewise, this study utilized the same standardized clinical examination and questionnaires at base line and at follow-up. Two recently released pediatric versions of DC/TMD have been published, covering ages 6-9 years and adolescents. However, these versions were not available at the start of the present study but should be considered in future studies [61, 62].

Today, JIA patients are very well taken care of in general regarding symptoms and inflammatory activity. Biological drugs are readily available and there is much evidence-based knowledge regarding diagnosis and treatment regimens [3, 63, 64]. This means that most JIA patients can be treated to a low or irrelevant systemic inflammatory activity and their prognosis is usually good regarding joint damage and pain [65]. In the present study, the patients had in general few orofacial signs and symptoms at the same time as they were adequately medicated. However, the patients were representative of the Swedish cohort of JIA patients regarding sex distribution, subtype variation and pharmacological interventions [30].

Clinical signs and symptoms as well as psychosocial factors were assessed with a two-year interval. The influence of the two-year difference between the assessments in these young patients on how they responded to the same questions is very difficult to assess but may play a role in the differences seen in our study. A two-year mental development in the ages included in this study can possibly make a difference. In addition, the interaction between the parents and the child may possible also affect the patient response but this was not assessed in the current study. However, two of the instruments used have been validated for use in the age range and all of them have been used in studied including children.

## Conclusions

This study indicates the importance of maintaining a low stress level in patients with JIA since an increase in stress level over a two-year period impairs jaw function as well as psychological distress and catastrophizing.

## Data Availability

The data underlying this article will be shared on reasonable request to Alexandra D. Carlsson at alexandra.carlsson@regionostergotland.se.

## Abbreviations

CHAQ: Childhood Health Assessment Questionnaire
DC/TMD: Diagnostic Criteria for Temporomandibular Disorders
GCPS: Graded Chronic Pain Scale
ILAR: International League of Association for Rheumatology
JADAS: Juvenile Arthritis Disease Activity Score
JFLS: Jaw Function Scale
JIA: Juvenile Idiopathic Arthritis
PCS: Pain Catastrophizing Scale
PHQ: Patient Health Questionnaire
PSS: Perceived Stress Scale
TMD: Temporomandibular Disorders
TMJ: Temporomandibular Joint
